# Genomic Landscape of Copy Number Variations and Their Associations with Climatic Variables in the World’s Sheep

**DOI:** 10.3390/genes14061256

**Published:** 2023-06-13

**Authors:** Hosein Salehian-Dehkordi, Jia-Hui Huang, Nasrollah Pirany, Hossein Mehrban, Xiao-Yang Lv, Wei Sun, Ali Esmailizadeh, Feng-Hua Lv

**Affiliations:** 1 College of Animal Science and Technology, China Agricultural University, Beijing 100193, China; hosein_salehi6@yahoo.com (H.S.-D.); s20213040574@cau.edu.cn (J.-H.H.); 2 Department of Animal Science, Faculty of Agriculture, Shahrekord University, Shahrekord 88186-34141, Iran; napirany@yahoo.com (N.P.); hosseinmehrban@gmail.com (H.M.); 3 Joint International Research Laboratory of Agriculture and Agri-Product Safety of Ministry of Education of China, Yangzhou University, Yangzhou 225009, China; dx120170085@yzu.edu.cn (X.-Y.L.); dkxmsunwei@163.com (W.S.); 4 International Joint Research Laboratory in Universities of Jiangsu Province of China for Domestic Animal Germplasm Resources and Genetic Improvement, Yangzhou University, Yangzhou 225009, China; 5 Department of Animal Science, Faculty of Agriculture, Shahid Bahonar University of Kerman, Kerman 76169-14111, Iran

**Keywords:** sheep, CNVs, climate adaptation, association tests, solar radiation

## Abstract

Sheep show characteristics of phenotypic diversity and adaptation to diverse climatic regions. Previous studies indicated associations between copy number variations (CNVs) and climate-driven adaptive evolution in humans and other domestic animals. Here, we constructed a genomic landscape of CNVs (*n* = 39,145) in 47 old autochthonous populations genotyped at a set of high-density (600 K) SNPs to detect environment-driven signatures of CNVs using a multivariate regression model. We found 136 deletions and 52 duplications that were significantly (*P*_adj._ < 0.05) associated with climatic variables. These climate-mediated selective CNVs are involved in functional candidate genes for heat stress and cold climate adaptation (e.g., *B3GNTL1*, *UBE2L3*, and *TRAF2*), coat and wool-related traits (e.g., *TMEM9*, *STRA6*, *RASGRP2*, and *PLA2G3*), repairing damaged DNA (e.g., *HTT*), GTPase activity (e.g., *COPG*), fast metabolism (e.g., *LMF2* and *LPIN3*), fertility and reproduction (e.g., *SLC19A1* and *CCDC155*), growth-related traits (e.g., *ADRM1* and *IGFALS*), and immune response (e.g., *BEGAIN* and *RNF121*) in sheep. In particular, we identified significant (*P*_adj._ < 0.05) associations between probes in deleted/duplicated CNVs and solar radiation. Enrichment analysis of the gene sets among all the CNVs revealed significant (*P*_adj._ < 0.05) enriched gene ontology terms and pathways related to functions such as nucleotide, protein complex, and GTPase activity. Additionally, we observed overlapping between the CNVs and 140 known sheep QTLs. Our findings imply that CNVs can serve as genomic markers for the selection of sheep adapted to specific climatic conditions.

## 1. Introduction

The earliest domestic sheep were domesticated in the Fertile Crescent [1,2]. Following domestication, they diffused worldwide, and various breeds with diverse phenotypes have been developed under long-term artificial and natural selection in response to human demands and climate change [3,4,5,6]. Climate-mediated genetic variations in the genome indicate that environmental factors, such as sunshine, temperature, elevation, and humidity, have influenced the spatial distribution of phenotypic and genetic variation across populations [5,7,8,9,10].

Copy number variations (CNVs), complementary to single nucleotide polymorphisms (SNPs) [11], are a major source of variations caused by deletions and duplications. They account for 4.8–9.5% of the whole genome and contribute to the variability in the genome among individuals [12,13]. Copy number variants (CNVs) can generate meiotically and somatically [14] and play an essential role in the rapid evolution process by changing the expression levels of genes with variable copy numbers [15,16] and dosage [17,18]. CNV of the *ASIP* gene regulates goats’ white and grey coat phenotypes [19]. Duplication in the flanking of *KIT* gene is associated with a white coat color in pigs [20]. CNVs identified by genome-wide association studies and selective sweep analyses in sheep have been related to complex traits, such as follicular development and fertility, milk production, wool production, adipogenesis, spleen size, and oxygenated red blood cells [21,22].

Climate-mediated selective pressures impact species distributions, phenotypic variation, and allele frequencies [23]. However, various climate factors, such as temperature, precipitation, altitude, and sunlight, may have distinct impacts on the level of genetic variation among species, influencing their potential for genetic adaptation [5,24]. For example, signatures of local genetic adaptation in genes are related to GTPase activities and energy metabolism caused by mutagenic factors sunlight [5], and positive selection genes with hypoxia responses were enriched in O_2_/CO_2_ exchange and HIF-1 signaling pathways by increasing the supply of O_2_ or reducing the harm of low oxygen saturation level in sheep [8]. Recently, solar ultraviolet (UV) radiation, as a highly mutagenic factor, can hinder DNA replication and transcription with photo-dimers, oxidative DNA lesions, and DNA single-strand breaks [24], and has been uncovered to influence variations of CNVs in frequency and structure due to sequence permutations in the molecular evolution of DNA [24,25,26]. Environmental factors, such as vinclozolin, contribute to the transgenerational inheritance of epigenetic modifications in sperm, leading to genome instability. This instability further promotes the acquisition of genetic copy number variations (CNV) in later generations [27].

It was demonstrated that CNVs are involved in adaptive evolution under various climates within diverse species, such as humans [28], cattle [29], goats [30], horses [31], and dogs [32]. CNVs of *AMY1* and *AMY2B* genes in humans and dogs are associated with adapting to the digestion of starchy foods [33,34]. *TAS2R16* is involved in gustation, and CNVS of the genes in cattle may be related to adapting to food resources and vegetation diversity [35]. CNVs in goats enriched in the IMAP family genes may play a role in adaptation to hash climates by regulating metabolism [36]. CNVs identified in Tibetan horses overlapped with genes (e.g., *CYP4A11*, *CYP4X1*, *EIF2AK1*, *CYP2C18*, *CYP4F22*, *NOS2*, and *CYP4B1*), which may account for adaptation to high altitude environment [37]. Climate change poses a significant threat to animal husbandry and food safety. A great deal of evidence illustrates that organisms can respond to climate change through phenotypic plasticity and evolutionary adaptation [38]. However, the challenge is identifying variations contributing to climate adaptation across the genome million variations. Nevertheless, a study has yet to be conducted to investigate the associations between CNVs and climatic variables during the post-domestication expansion of sheep. In recent years, the availability of the sheep reference genome of *Ovis aries* (Oar_v4.0) and a comprehensive set of high-density (600 K) single nucleotide polymorphisms (SNPs) provided the opportunity to investigate the contribution of CNVs to rapid local climatic adaptation in autochthonous sheep populations [5,9,39]. Here, we performed unified co-analyses of CNVs, and environmental variables were generated from a global climatic data set of 117 parameters over 40 years in 47 autochthonous sheep populations. We examined the impact of solar radiation on the genomic distribution of CNVs in sheep of different geographic origins.

## 2. Materials and Methods

### 2.1. Data Collecting, CNV Calling, and Quality Controls

**Figure 1:**
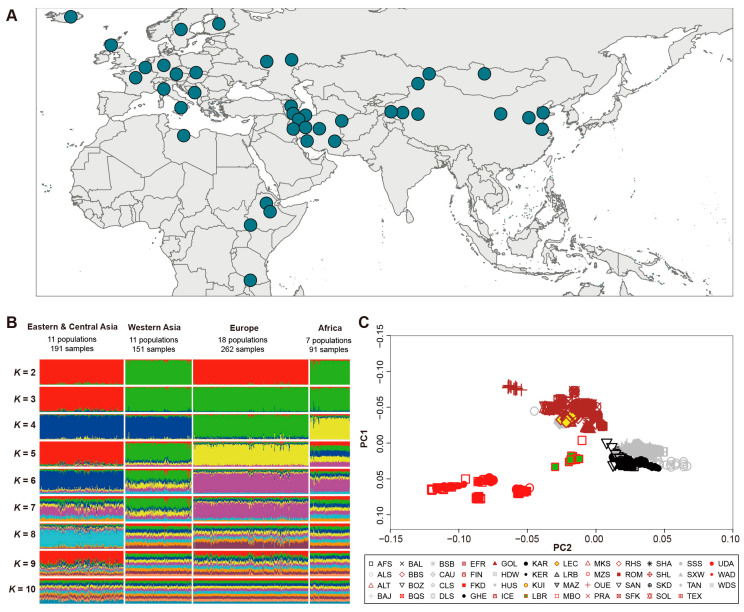
Population genetic structure. (**A**) Geographic origin of 47 worldwide autochthonous sheep populations. (**B**) Population genetic structure of 47 autochthonous sheep populations (*K* = 2–10). (**C**) Principal component analysis (PCA) of 47 autochthonous sheep populations. The brown, red, gray, and black symbols represent Europe, Africa, Eastern-Central Asian, and Western Asian populations.

We collected the Ovine Infinium HD (600 K) SNP Bead Chip data from previous studies [9,39]. We upgraded all the positions in the SNP BeadChip based on the *O. aries* reference genome Oar_v.4.0 (https://www.ncbi.nlm.nih.gov/assembly/GCF_000298735.2, last accessed on 24 September 2020). To exclude poor-quality DNA samples and SNPs, we only used SNPs that passed the filtering procedure described in Salehian-Dehkordi et al. [39]. Additionally, any SNPs on the X and Y chromosomes were excluded. After quality control and filtering, 47 worldwide autochthonous populations, including 695 individuals (Supplementary Table S1 and Figure 1A), were selected for the following analyses. We detected CNVs and CNVRs (copy number variable regions) using the approach described previously [39]. In summary, we first retrieved each sample’s signal intensity values (log R ratio: LRR; B allele frequency: BAF) by the Illumina GenomeStudio v1.0. Second, the B allele matrix population frequency (PFB) was calculated with the script compile_pfb.pl using PennCNV v1.0.53 [40]. Third, we detected CNVs with the same parameters as Salehian-Dehkordi et al. [39]. Finally, the raw CNVs were filtered with the following criteria: (i) individual with a standard deviation of LRR < 0.3; (ii) BAF drift of individual < 0.01; (iii) waviness factor of individual < 0.05; (iv) CNVs with more than 3 consecutive SNPs and at least 1 kb length; (v) CNVs with call count > 100 per sample, which represents poor DNA quality, and relevant individuals were removed. We merged all CNVs into CNVRs using bedtools version 2.30.0 (https://bedtools.readthedocs.io/en/latest/index.html, accessed on 23 January 2021) with the “bedtools merge” option [41].

### 2.2. Environmental Data

We accessed climatic data covering a period of 40 years (1961 to 2001) from the global climate data set (http://www.cru.uea.ac.uk/data, last accessed on 24 September 2021) (The Climatic Research Unit, Norwich [42]). The climatic data contained yearly and monthly means of nine parameters, including (i) average daily duration of bright sunshine in percent (sunshine fraction, SUN); (ii) mean diurnal temperature range in °C (DTR); (iii) relative humidity in percentage (REH); (iv) precipitation in mm/month (PR); (v) mean temperature in °C (TMP); (vi) the coefficient of variation of monthly precipitation in percent (PRCV); (vii) number of days with ground frost (FRS); (viii) 10 m wind speed in m/s (WND); and (ix) number of days with >0.1 mm rain per month (RDO) (Supplementary Table S1). In total, 117 environmental variables and elevation for the geographic origins of 47 autochthonous populations were obtained based on the Latitude (°N) and Longitude (°E) coordinates (Supplementary Table S1, Supplementary Material online, in Cao et al. 2021 [9]).

### 2.3. Estimation of Solar Irradiation

We have extracted the solar radiation from the sunshine fraction (SUN variable) using the relationship between sunshine and radiation described in Suehrcke et al. [43]. We first obtained annual average sunshine fractions for the locations of interest from Cao et al. [9]. Next, the annual average extraterrestrial radiation was calculated from average monthly values for each location (see Supplementary Tables S2 and S3) based on the following equation [44]:

$${\bar{H}}_{o}=\frac{86,400\;G_{sc}}{\pi }\left[1+0.033\;cos\left(2\pi \frac{n}{365}\right)\right]\times (\cos\Phi \cos\Delta \sin\omega_{s}+\omega_{s}\sin\Phi \sin\Delta )$$

where ${\bar{H}}_{o}$ is the monthly average of extraterrestrial daily solar radiation on the earth’s surface without atmosphere (extra-terrestrial radiation) [MJ/m^2^/day], $G_{sc}$ is the solar constant (the latest suggested value is 1361 W/m^2^ [45]), *π* is approximately 3.14159, *n* is an average day of month, Φ is latitude (North +, South −), Δ is declination: $\Delta =\left(23.45\frac{\pi }{180}\right)sin\left(2\pi \frac{284+n}{365}\right)$ [44], and $\omega_{s}$ is the solar hour angle at sunset: $\omega_{s}=acos\left(-\tan\Phi \times \tan\Delta \right)$.

After determining the annual sunshine fraction and extraterrestrial radiation, the annual average clearness index $\bar{K}$ was computed to estimate the annual average solar radiation in geographic origins of autochthonous sheep breeds. $\bar{K}$ can be expressed as:

$$\bar{K}={\bar{K}}_{clear}\left(\beta +(1-\beta )\times S^{\gamma }\right)$$

where $\bar{K}$ is the annual clearness index, ${\bar{K}}_{clear}$ is the approximate world average clear sky clearness index value (0.7191), β and *γ* are constants 0.1930 and 0.7283, respectively, and S is the sunshine fraction for the different sampling locations from Cao et al. [9] (see Supplementary Table S1). Finally, we calculated the annual average daily solar radiation ($\bar{H})$ for each sampling site using the equation $\bar{H}$ = $\bar{K}$ × ${\bar{H}}_{o}$ (see Supplementary Table S3).

### 2.4. Testing for Genomic Signatures Associated with Climate Variables

Two approaches were used to detect the signatures of local adaptation. We first used multiple univariate logistic regression models [46] to detect genotype–environment associations under individual-based analysis. We analyzed using the Samβada program (https://lasig.epfl.ch/sambada, last accessed 24 September 2020) by incorporating population structure to decrease the occurrences of false genotype–climate relationships under the “*BEST*” option. For the analysis, a specific CNV state of all related samples was marked as either present or absent (i.e., binary information: 1 or 0). Correlations between all possible copies of CNV events and climatic variables were estimated across the sampling locations, and only significant models by Bonferroni correction based on the Wald statistic (*p* < 0.01) were considered.

We further applied latent factor mixed models to detect associations between CNVs and environmental variables based on population genomic statistics [47]. The methods incorporated fixed, random, and latent effects (i.e., demographic history of autochthonous breeds and isolation-by-distance patterns) to lower the risk of false-positive associations in landscape genomics [47]. We first summarized all the climatic factors and elevation using the first axis of the PCA to reduce the dimensionality of multivariate data into two columns of principal components with minimal loss of information. We used the ade4 package to perform the PCA analysis for individuals/variables. Following that, the latent factor (*K* = 4) was identified based on the distribution of populations in structure analyses using Structure v2.3.4 [48] and SmartPCA [49]. For each CNV, we calculated the latent score (*Z* scores) based on the least-squares estimation approach [50]. A threshold (|*Z*| scores ≥ 10) was used to identify significant CNV effects at a level of *p* < 10^−6^ after Benjamini-Hochberg correction for multiple testing with 5% type I error [47,50]. We conducted the analysis using latent factor mixed models version 2 (LFMM2) (https://rdrr.io/bioc/LEA/man/lfmm2.html, accessed on 13 September 2022), as implemented in the R package LEA [50].

### 2.5. Testing for Genomic Signatures Associated with Solar Radiation-Mediated Selective Pressure

Using probe-based statistics, we performed genome-wide association analyses of CNVs with solar radiation variables. CNVs and quality measures were converted to matrices containing probes for deletions and duplications separately using ParseCNV v2 [51] under the parameter “*-includePed*”. We implemented two approaches to perform probe-based association regression tests. First, we calculated the probe-based statistical significance of neighboring SNPs using the EMMAX program (http://csg.sph.umich.edu//kang/emmax/download/index.html, accessed on 7 March 2010) [52]. We constructed kinship matrices of BN (Balding-Nichols) using the EMMAX algorithm and the script “*emmax-kin-intel64*”. The association between probe-based statistics for CNV occurrence and solar radiation was computed using mixed linear models with the “*emmax-intel64*” argument. To correct for population variation, the PCs as covariates were implemented simultaneously in the model using “*-c*” and “*cov_file*” options. A suggestive threshold (*p* < 5 × 10^−4^) was considered for probe-based statistical significance of CNV occurrence [51], and *p* values were computed using the EMMAX method.

Next, we tested the associations between probes of deleted/duplicated CNVs and solar irradiance using the PLINK v1.90 in the ParseCNV environment. Multi-covariate association analyses were applied for the logistic regression models using the codes “*-logistic*” and “*-adjust*” arguments, also “*-covar*” option was run to implement PCA population stratification components. Finally, we used the script “*InsertPlinkPvalues.pl*” in the ParseCNV program to merge adjacent probe-based CNV occurrence *p*-values into significant CNVRs. The code “*-permuteP <* 10,000 *>*” was run with 10,000 permutations in the algorithm. We implemented a significance threshold of max (T) adjusted (*p* < 0.05) after multiple testing corrections to define CNVRs and therefore reported associations. To evaluate significant CNVRs for confidence, the program ParseCNV amassed red flags based on various factors referenced in the University of California Santa Cruz (UCSC) browser. These characteristics help to reduce false positive CNV detection based on the criteria such as segmental duplications, the database of genomic variants, centromere/telomere, GC base content, density and count of probes, length of the CNVs, population frequency, peninsula, and inflated (Supplementary Figure S2) [51].

### 2.6. Gene Annotation and Overlapping with QTLs

We annotated the gene content associated with the candidate CNV and CNV regions using the sheep reference genome Oar_v.4.0. Only genes that overlapped with a CNVR spanning at least 10% of the CNV length were considered. We implemented Gene Ontology (GO) and Kyoto Encyclopedia of Genes and Genomes (KEGG) pathway analyses using the DAVID (database for annotation, visualization, and integrated discovery) [53]. Categories with adjusted *p* value < 0.05 after the Bonferroni correction and at least 12 genes were defined as significantly enriched GO terms and KEGG pathways.

We further annotated the function of the candidate CNV and CNV regions with quantitative trait loci (QTLs). We identified overlapping regions among sheep QTLs and the candidate CNV and CNV regions. The overlapping regions were detected using Bedtools v 2.30.0 under the setting: (i) QTLs with confidence interval < 5 Mb; (ii) CNVR spanning at least 50% of the CNV length [54].

## 3. Results

### 3.1. CNV Detection and Population Differential Analyses

**Figure 2:**
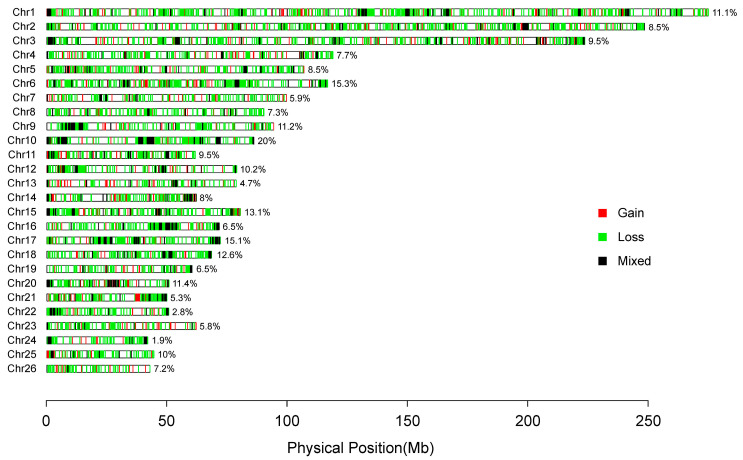
CNVR distribution in autochthonous sheep populations on each chromosome.

In total, 129,446 raw CNVs were identified from 695 samples by PennCNV v1.0.53. After quality controls, 39,145 high-quality CNVs were obtained and merged into 4769 CNVRs based on the overlapping region among CNVs (Table 1, Supplementary Table S4, Figure 2). To determine the accuracy of CNVs, we validated all CNVs by comparing CNVs identified in this study with CNVs in Salehian-Dehkordi et al. [39]. The genotyping concordance was 57.34% (Supplementary Figure S1). We used two approaches (e.g., Structure and PCA analyses) to uncover population features based on CNV frequencies. We observed four genetic clusters corresponding to four geographic regions (Figure 1B,C). All the 47 native breeds were clustered into four groups based on genetic and geographic features: (i) 11 populations (*n* = 191) from Eastern and Central Asia; (ii) 11 populations (*n* = 151) from Western Asia; (iii) 7 population (*n* = 91) from Africa; and (iv) 18 populations (*n* = 262) from Europe (Figure 1).

### 3.2. Function Annotation of CNVRs

A total number of 2892 genes were annotated within 4769 CNVRs in 47 autochthonous sheep populations (Supplementary Table S5). We further investigated the functions of those genes by two methods. Firstly, we used GO term and KEGG pathway enrichment analyses. We detected nine significant (*P*_adj._ < 0.05) Gene Ontology categories (Supplementary Table S6), including protein complexes in cytoplasm (GO:0005829, *p* = 1.91 × 10^−8^ and GO:0005737, *p* = 4.4 × 10^−5^), collagen trimer (GO:0005581, *p* = 5.31 × 10^−5^), nucleoplasm (GO:0005654, *p* = 0.01268), ATP binding (GO:0005524, *p* = 3.05 × 10^−9^), GTPase activator activity (GO:0005524, *p* = 1.71 × 10^−5^), microtubule binding (GO:0008017, *p* = 6.5 × 10^−4^), calcium ion binding (GO:0005509, *p* = 9.51 × 10^−4^), and guanyl-nucleotide exchange factor activity (GO:0005085, *p* = 0.0047). Eleven categories of KEGG with the threshold of adjusted *P*_adj_ value < 0.05 after the Bonferroni correction were identified, such as axon guidance (oas04360, *p* = 5.07 × 10^−6^), focal adhesion (oas04510, *p* = 5.00 × 10^−4^), and calcium signaling pathway (oas04020, *p* = 0.038) (Supplementary Table S6). Secondly, we annotated the 4769 CNVRs and detected 80 unique CNVRs overlapped with 140 QTLs (Supplementary Tables S7 and S8). Of these QTLs, 25 overlapped QTLs for the disease resistance traits, six for reproductive traits, 38 for body size and meat and growth-related traits, 12 for milk-related traits, and four for wool-related traits.

### 3.3. Climate-Driven Candidate Selective Signatures Testing

**Figure 3:**
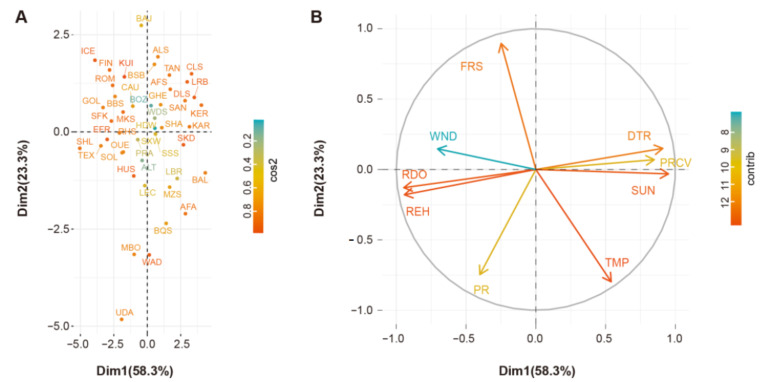
PCA of environmental variables. (**A**) Projection of the nine climatic variables based on 47 worldwide autochthonous populations. The representation of the variable is quantified by cos2, and a high cos2 indicates a good representation of the variable on the principal component. (**B**) Projection of the nine climatic variables on the first and second factor planes. SUN: percent of maximum possible sunshine; DTR: mean diurnal temperature range in °C; REH: relative humidity in percentage; PR: precipitation in mm/month; TMP: mean temperature in °C; PRCV: the coefficient of variation of monthly precipitation in percent; FRS: number of days with ground frost; WND: 10 m wind speed in m/s; RDO: number of days with >0.1 mm rain per month. Contributions of variables to PCs are shown by the colors bar.

The Biplot showed PC1 versus PC2 for the 47 autochthonous sheep populations and the climatic parameters of their geographic origins. All groups of individuals (Figure 3A) and climatic variables (Figure 2B) were mapped using their contributions. The colors show qualities of representation for variables and populations on the maps.

We conducted the analysis by Samβada program and selected the top models according to the Wald statistics (*p* < 0.01 and Wald score > 34), and 377 out of a total of 4,579,965 univariate models (39,145 CNV genotype × 117 climatic variables) were selected. Thirty-four genes were annotated within 377 CNVs, and eight genes from 33 unique CNVs were associated with environmental variables (Supplementary Table S9), such as *ATHL1*, *NLRP6*, *IFITM5* for PR, and *GUCY1A2* for PRCV (Supplementary Table S10). Next, based on the z score in the latent factor mixed models (LFMMs), we detected 155 CNVs with |z| scores ≥ 10 (Supplementary Table S11) that were highly significant (3.63 × 10^−6^ ≥ *p* values ≥ 1.34 × 10^−103^), associated with environmental parameters in 47 old sheep populations. We then found genes that overlapped CNVs (e.g., *B3GNTL1*, *UBE2L3*, *TRAF2*, *GTF2F1*, and *IGFALS*), which were significantly associated with climatic variables (Supplementary Tables S10 and S12).

### 3.4. Solar Radiation-Driven Candidate Signatures Test

**Figure 4:**
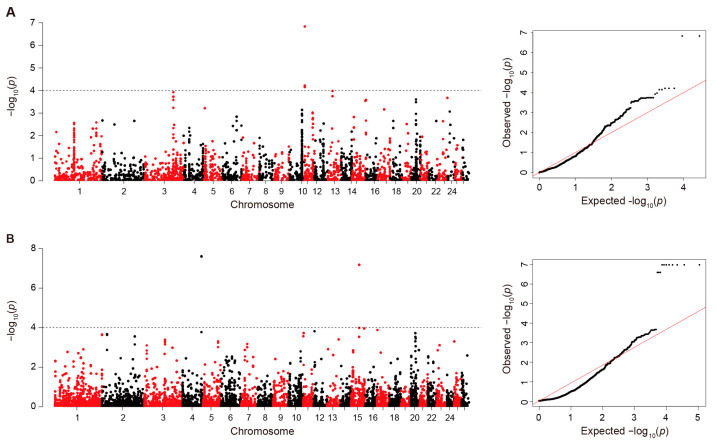
Manhattan plot of GWAS between probes in deleted/duplicated CNVs and solar radiation. (**A**) Manhattan plot based on duplicated CNVs; the horizontal dashed lines correspond to the genome-wide significance thresholds (*P*_adj._ = 6.08 × 10^−5^). (**B**) Manhattan plot based on deleted CNVs; the horizontal dashed lines correspond to the genome-wide significance thresholds (*P*_adj._ = 6.57 × 10^−8^).

We implemented association tests for solar radiation variables using the linear mixed models in the EMMAX program. To detect associated deletions and duplications, we found 54,597 and 13,925 probes among deletion and duplication regions for subsequent association tests, respectively. Among the identified probe-based statistics, we found 86 and 45 probes for deletions and duplications, respectively, based on genome-wide CNV significant threshold (*p* < 5 × 10^−4^) (see 5th columns in Supplementary Tables S13 and S14). Next, we detected significant associations through multi-covariate association analyses using the logistic regression models. We obtained the top significant (*p* < 5 × 10^−5^) SNP probes for deletions (180) and duplications (125) genomic regions among the full set of probes (see 9th columns in Supplementary Tables S13 and S14). We identified common significant probes detected at least by two approaches (EMMAX and ParseCNV-Plink). After Bonferroni correction in duplicated regions, seven significant probes on chromosome 11 with *p* ≤ 6.08 × 10^−5^ were associated with solar irradiance (Figure 4A). In deleted regions, three and eight probes on chromosomes 15 and 4 (*p* ≤ 6.57 × 10^−8^) were significantly associated with solar radiation, respectively (Figure 4B).

Based on the probe-based statistics for CNV occurrence detected by two approaches, we identified associations between CNVRs and solar irradiances. By applying the program ParseCNV, noisy CNVs were removed, and normalized results were implemented in the association test (Supplementary Figure S2B). Afterward, 35 significant CNVRs (adjusted *p* < 0.05) were associated with solar radiation (Supplementary Table S15), and some of them overlapped with functional genes (Table 2 and Supplementary Table S16). We found that CNVs and CNVRs were commonly selected by statistical tests (Supplementary Table S17).

## 4. Discussion

High-density SNP data have been used to detect CNVs in livestock [21,55,56]. The genome-wide testing of CNVs for phenotypic trait variations was reported previously, but climatic adaptation involving CNVs still needs to be explored. This study showed the results of CNV genotypes called by the high-density SNP. We performed association tests between CNVs and climatic variables and detected candidate genes for environment-driven genetic adaptation. Unlike our previous study, which used only SNPs to find associations between alleles and climates [9], we applied ecological tests to find association signals between CNV states and environmental variables for the first time. We observed a set of novel candidate genes which overlapped with CNVs. They were significantly correlated with local climatic adaptation.

We found 39,145 high-quality CNVs, and the genotype of each CNV was confirmed by being compared with previous results in Salehian-Dehkordi et al. [39] (see Supplementary Tables S3 and S7 in Salehian-Dehkordi et al. [39]). The results showed a high level of genotypic concordance between them (57.34%) (Supplementary Figure S1). We found that the frequency of hemizygous (59.52%) and homozygous deletions (12.07%) was more than duplications (28.15%) and monoallelic triplications (0.26%) (Table 1 and Supplementary Table S4), and the results agree with previous reports [57,58,59,60].

As an essential source of polymorphism in genomes, CNVs might account for phenotypic variations [61,62]. Here, we found 2892 genes within CNVRs (Supplementary Table S5). Functional enrichment analysis showed that genes in CNVRs are associated with physiological functions, energy metabolism, nervous system, immunity, and phenotype (Supplementary Table S6). Of the genes, *MLPH* located in chr1:3417932-3436288 encodes the melanophilin carrier protein involved in pigmentation in the hair and skin (Supplementary Table S5) [63]. Interestingly, a hemizygous deletion (chr1:3.112.486-3122600) neared downstream of the mentioned CNVR was detected by the LFMM analysis. We also found other genes related to pigmentation or defense against pathogens, such as *TYR*, *LPO*, and *REN*, which may be involved in local adaptation [64,65,66].

Our results suggested that autochthonous sheep breeds’ adaptation to climates could be partly ascribed to CNVs, as found in organisms such as cattle [67] and balsam poplar [57]. The multiple univariate logistic regression analyses found three critical functional genes on chr21:49691288-49714065 (Supplementary Table S10). CNV harboring functional candidate gene (*OAS2*) with a high Wald score was associated with fat deposition in sheep breeds [68]. The tail fat might benefit adaptation to extreme environments and harsh seasons [69]. These results suggested that the genes with different numbers of copies are under climate-mediated selective pressures.

The LFMM analysis further supported the idea that CNVs mediate the genetic response of autochthonous sheep breeds to climates. We explored the involvement of hemizygous deletions resulting from evolutionary climatic adaptation pressure (Supplementary Table S11). Those hemizygous deletions harbored functional genes, such as *B3GNTL1*, *UBE2L3*, *TRAF2*, *TF2F1*, and *IGFALS*. *B3GNTL1* gene identified here has been reported to regulate heat stress adaptation in Egyptian sheep breeds [70]. Additionally, ubiquitin-conjugating enzyme E2-L3 (*UBE2L3*) was involved in response to heat stress in Indian cattle [71]. *TRAF2* and *GTF2F1* (a duplicated gene) genes were identified in Russian sheep breeds, Chinese cattle, and Datong yak populations, indicating their associations with adaptation to high altitudes and cold climates [72,73,74]. Together, these results suggested the involvement of CNVs in generic and environmental adaptation.

We found two common CNVRs on chromosome 5 identified by LFMM and Samβada analysis (Supplementary Table S18). Four common significant probes were detected in climatic and solar radiation-driven selective pressure testing (Supplementary Table S18). The result indicates potential complex and specific genetic mechanisms responding to environmental factors.

To find significant associations between CNV-associated genes and solar radiation, we merged probe-based statistics from CNV calls into CNVRs (Supplementary Table S16). Interestingly, we identified candidate genes such as *HTT* that play an important role in repairing damaged DNA [75], *RUVBL1* for heat and parasite stress [76], *SF1* and *PHLDA2* for litter size and reproduction [29], *SLC41A3* for immune responses [77], and *KLF15* for growth-related traits [78] (Supplementary Table S17). *HTT* is important in repairing DNA damage created by solar and UV radiation. In addition, a cascade of physiological events will be initiated when an animal is exposed to solar radiation [79,80]. For example, an endogenous circannual rhythm driven and synchronized by the annual photoperiod cycle regulates the breeding season in sheep [81,82]. UV radiation and climatic factors, including temperature and humidity, can also affect the spread of pathogens in mammals [83,84].

Many selected CNV-overlapping genes via ecological tests were associated with production traits and immunity (Supplementary Table S19), suggesting that these CNV-associated genes might be signatures of natural and artificial selections in their adaptation to extreme climates. Our results supported the hypothesis of the 119 selective functional genes associated with climate-mediated selection—21 genes (e.g., *STRA6*, *TMEM9*, and *PLA2G3*) were responsible for coat and wool, 20 genes (e.g., *B3GNTL1*, *TRAF2*, and *GUCY1A2*) for environmental condition and stress, 15 genes (e.g., *LMF2*, *LPIN3*, *COPG1*, and *OAS2*) for metabolism, including GTPase and lipid metabolism activities, 14 genes (e.g., *NPBWR2*, *OAS1*, and *SLC19A1*) for fertility and reproduction, 18 genes (e.g., *SFTPD*, *RNF121*, and *TMEM154*) for immunity, cancer, and disorders, and 28 genes (e.g., *KLF15*, *CACNA1S*, and *CCDC152*) for growth and production traits (Supplementary Table S19). We observed some candidate genes (e.g., *LTN1*, *TRAF2*, *COPG1*, and *SLC19A1*) overlapped with candidate CNVs in sheep detected previously [74,85,86]. Additionally, some functional genes were previously detected as related to climate-mediated livestock adaptation [5,9].

Previous reports showed the role of important CNVs for specific traits such as coat color [87]. A considerable number of selective genes from association tests, by the LFMM approach, were associated with coat and wool traits in livestock (e.g., *MEGF6*, *STRA6*, *LTN1*, *FAM83G*, and *TRAF2*) (Supplementary Table S12). These genes were under natural selection due to the high-altitude adaptation or the intensive UV and solar radiations under harsh environments, such as the Tibetan plateau [88].

Genotype-by-environment interactions for production traits in beef cattle studies indicated the role of the biological system (e.g., vasodilation, metabolism, and nervous) in the genetic sensitivity to environmental stress [89,90]. In agreement with previous studies, we found many significant CNVs that overlapped genes (e.g., *PCDH15*, *MAPK1*, *DNAJB8*, and *SHANK2*) related to heat stress and metabolism (Supplementary Table S19). Of the common CNVs and CNVRs obtained from more than one association test (Supplementary Table S18), *INPP5A* and *KLF15* were related to heat stress and production traits, respectively [78,91]. Our results indicated that genotype-by-environment interactions could have contributed to copy number status during adaptation to different environmental stress.

## 5. Conclusions

This is one of the first comprehensive CNV studies to reveal novel associations with environmental variables. Overall, 39,145 high-quality CNVs were identified in the worldwide sheep populations, which harbored 2892 genes associated with phenotypic traits and climatic-mediated adaptive variations. We demonstrated evidence for the climate-mediated genes in CNVRs. This study indicated the genes with different numbers of copies retrieved from natural selection for specific traits such as heat stress, cold adaptation, and metabolism. CNV harbors specific functional candidate genes such as *B3GNTL1*, *UBE2L3*, *SHANK2*, *COPG1*, *TRAF2*, and *GTF2F1* for heat stress and cold climate adaptation *LTN1*, *STRA6*, *RASGRP2*, and *HTT* for repairing damaged DNA. This study generated a CNV map and revealed important candidate genes associated with climate adaptation.

## Figures and Tables

**Table 1:** Summary of CNVs and CNVRs identified in old autochthonous sheep populations.

Type	CNVs					CNVRs				Unique CNVs			
	Homozygous Deletion	Hemizygous Deletion	Duplication	Biallelic Triplication	Total	Loss	Gain	Mixed	Total	Deletion	Duplication	Mixed	Total
Count	4726	23,298	11,018	103	39,145	3468	861	440	4769	11,119	4118	510	15,747
Length (Mb)	94.4	801.2	460.9	3.2	1359.7	150	23.4	80.9	254	492.6	213.6	14.2	720.4

**Table 2:** The significant CNVRs and candidate genes associated with solar radiation under adaptive selection.

Significant CNVRs	Count SNPs	Gene ID
chr16:60477356-60501528	9	*LOC101104428*, *LOC101120862*
chr16:69228726-70108247	26	*IRX1*, *LOC105602651*, *LOC105602652*, *LOC105608090*
chr26:399178-402542	4	*DLGAP2*
chr12:77533856-78344379	14	*LOC105610048*, *LOC105616624*, *LOC105610045*, *LOC105610042*, *LOC105610043*, *LOC106991502*, *RABIF*, *LOC106991503*, *LOC106991504*, *UBE2T*, *C12H1orf106*, *LOC105610049*, *CACNA1S*, *TMEM9*, *IGFN1*, *PKP1*, *LOC101109820*, *KLHL12*, *LOC105616599*, *SYT2*, *LOC105616625*, *LOC101110351*, *LOC101110859*, *KIF21B*
chr19:58987471-60169432	29	*LOC105603604*, *GATA2*, *DNAJB8*, *LOC105603605*, *LOC105603606*, *SEC61A1*, *LOC106991760*, *LOC105603608*, *LOC105603609*, *LOC105603631*, *LOC106991788*, *RPN1*, *EEFSEC*, ***RUVBL1***, *MGLL*, *ZXDC*, ***SLC41A3***, *ALDH1L1*, *MCM2*, *KBTBD12*, *PLXNA1*, ***KLF15***, *RAB7A*
chr21:41984078-42773808	4	*SLC22A12*, *MEN1*, *PPP2R5B*, *CDC42EP2*, *GPHA2*, *ZFPL1*, *TMEM262*, *TM7SF2*, *ZNHIT2*, *FAU*, *MRPL49*, *SYVN1*, *SPDYC*, *LOC101107147*, *TIGD3*, *SLC25A45*, *SLC22A11*, *LOC105604109*, *RASGRP2*, *LOC105604110*, *MAP4K2*, *CDC42BPG*, *EHD1*, *C21H11orf85*, *BATF2*, *NAALADL1*, *CDCA5*, *VPS51*, *LOC101105958*, *LOC105604111*, *SLC22A20*, *POLA2*, *DPF2*, *NRXN2*, ***SF1***, *ATG2A*, *SNX15*, *SAC3D1*, *ARL2*, *CAPN1*, *MIR194*, *PYGM*
chr21:46919932-47922324	17	*LOC105604138*, *LOC101118574*, ***PHLDA2***, *LOC105604139*, *DHCR7*, *NADSYN1*, *OSBPL5*, *CARS*, *NAP1L4*, *SLC22A18*, *LOC101118066*, *SHANK2*, *CDKN1C*
chr6:114803668-114941025	22	*MSANTD1*, *RGS12*, ***HTT***
chr14:55268932-55284965	7	*IZUMO2*, *LOC101115729*
chr17:70557208-70575800	7	*LOC106991688*, *GSTT2B*, *LOC101111397*, *LOC101118990*
chr13:53886521-53904177	9	*SLCO4A1*
chr16:31746166-31765064	6	*CCDC152*
chr1:262818152-263558472	31	*LOC105604794*, *POFUT2*, *SLC19A1*, *PCBP3*, *ADARB1*, *COL18A1*
chr14:48269630-48282740	4	*EID2*
chr22:49712254-50377777	25	*LOC105604373*, *PWWP2B*, *LOC105606161*, *LOC105604355*, *JAKMIP3*, *DPYSL4*, *LOC101109500*, *STK32C*, *INPP5A*, *LOC101110287*
chr3:137339004-137374322	8	*LOC101123028*, *LOC101123287*
chr18:64006157-64006658	3	*BEGAIN*

## Data Availability

The original contributions presented in the study are included in the article/Supplementary Material, further inquiries can be directed to the corresponding author.

## Supplementary Materials

The following supporting information can be downloaded at: https://www.mdpi.com/article/10.3390/genes14061256/s1.
