# Supplementary material for: Genomic Landscape of Copy Number Variations and Their Associations with Climatic Variables in the World’s Sheep
Source: Genes (Basel). 2023 Jun 13;14(6):1256. doi: 10.3390/genes14061256 (PMC10298528; doi:10.3390/genes14061256)
Supplement: Supplementary file 1 [file genes-14-01256-s001.zip › Supplementary Figures.pdf]

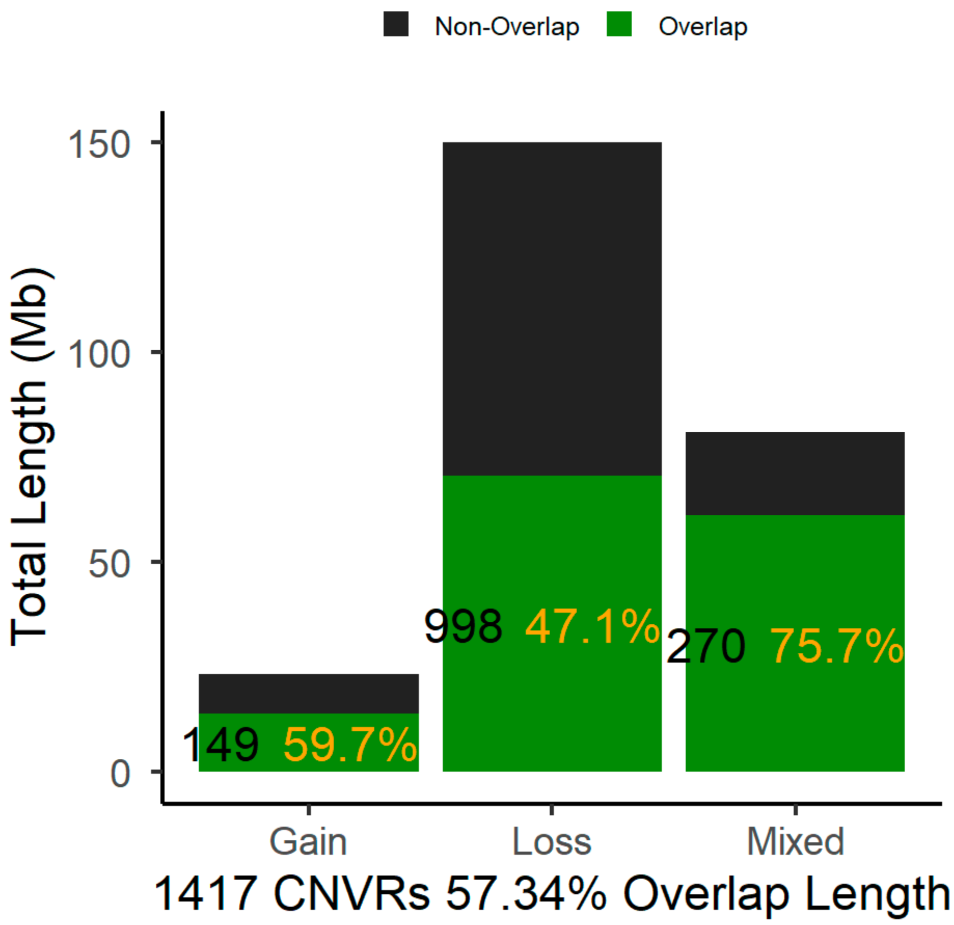

Supplementary Figure S1 Genotyping concordance between CNVs called from the study and previous study. The percentage shows genotyping concordance between this and Salehian-Dehkordi et al. (2021) studies on the population level.

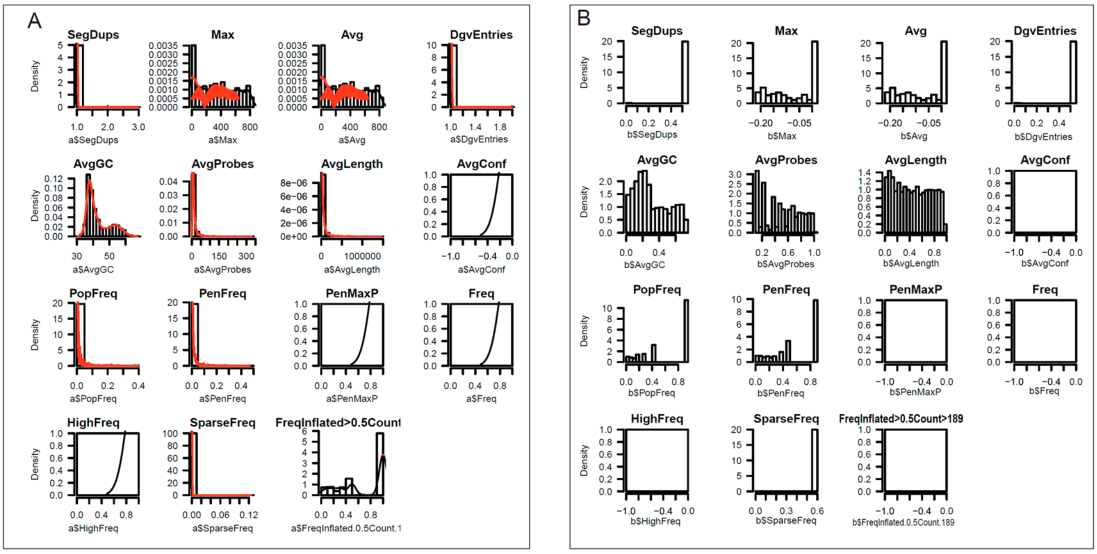

Supplementary Figure S2 (A) Confidence red flags histograms. From left to right the Y-axes are density of each parameter and X-axes explain segmental duplications (SegDups) for count, maximum (Max) and average (Avg), respectively; overlapping multiple database of genomic variants (DGV) entries; average GC base content (AvgGC); average number of probes (AvgProbes); average length of the CNVs (AvgLength); average confidence of CNVs captured with low number of probes (AvgConf); population frequency (PopFreq), CNVRs with large population frequency show that probe clustering is likely biased because a high percentage of individuals with CNV applied in clustering definition; CNV peninsula frequency (PenFreq) is the most common false-positive result; PenMaxP is the worst p-value in the span of CNVs which are contributed to the significant CNVR; the frequency of this PenMaxP (Freq); the frequency of any non-nominally significant p-value with P>0.05 (HighFreq); sparse coverage with high gap within probe coverage exists in the detected CNVs (SparseFreq); the similar inflated sample driving several association signals for CNV (FreqInflated>0.5Count), certain individuals have many noisy CNVs arising in rare regions even with quality control for samples. (B) Confidence red flags normalized histograms. Default report thresholds for each histogram from left to right including >10 for SegDups (count), > 0.98 max fraction matching (Max and Avg); > 10 for DGV entries; 31 > GC > 60 for AvgGC; < 10 for AvgProbes; < 10 kb for AvgLength; < 10 for AvgConf; > 0.01 for PopFreq; CNV peninsula frequency (PenFreq) is the most

common false-positive result; > 0.5 for PenMaxP; > 0.5 for Freq; > 0.05 for HighFreq; > 50kb for SparseFreq; > 0.5 sids at the locus have > (maxInflatedSampleCount-2) occurrences among all significants for FreqInflated.
